# Bibliometrics and visual analysis of metformin and gut microbiota from 2012 to 2022: A systematic review

**DOI:** 10.1097/MD.0000000000036478

**Published:** 2023-12-15

**Authors:** Yang Shu, Weidong Li, Qiongying Hu, Daqian Xiong

**Affiliations:** a Department of Laboratory Medicine, Hospital of Chengdu University of Traditional Chinese Medicine, Chengdu, China; b College of Medical Technology, Chengdu University of Traditional Chinese Medicine, Chengdu, China; c Chongqing Key Laboratory of Sichuan-Chongqing Co-construction for Diagnosis and Treatment of Infectious Diseases Integrated Traditional Chinese and Western Medicine, Chongqing, China.

**Keywords:** bibliometric, emerging topics, gut microbiota, metformin, research hotspots and trends

## Abstract

**Background:**

Metformin is an old drug used for the treatment of type 2 diabetes mellitus and can play a variety of roles by regulating the gut microbiota. The number of research articles on metformin in the gut microbiota has increased annually; however, no bibliometric tools have been used to analyze the research status and hot trends in this field. This study presents a bibliometric analysis of publications on metformin and gut microbiota.

**Methods:**

We searched the Web of Science core collection database on June 8, 2023, for papers related to metformin and gut microbiota from 2012 to 2022. We used Microsoft Excel 2021, VOSviewer1.6.19, CiteSpace 6.2.4, and R software package “bibliometrix” 4.0.0 to analyze the countries, institutions, authors, journals, citations, and keywords of the included publications.

**Results:**

We included 517 papers, and the trend in publications increased over the last 11 years. The 517 articles were from 57 countries, including 991 institutions and 3316 authors, and were published in 259 journals. China led all countries (233 papers) and the most influential institution was the Chinese Academy of Sciences (16 papers). *PLOS ONE* (19 papers) was the most popular journal, and *Nature* (1598 citations) was the most cited journal. *Li* and *Kim* were the 2 most published authors (six papers each), and *Cani* (272 co-citations) was the most co-cited author. “Metabolites,” “aging,” and “intestinal barrier” were emerging topics in this field.

**Conclusions:**

This bibliometric study comprehensively summarizes the research trends and progress of metformin and gut microbiota, and provides new research topics and trends for studying the effects of metformin on gut microbiota in different diseases.

## 1. Introduction

Metformin, originally derived from a plant called “goat bean,” is a commonly used oral hypoglycemic drug in clinical practice. It has been used to treat type 2 diabetes mellitus (T2DM) for more than 60 years because of its low cost, good hypoglycemic effects, and few side effects. Through an in-depth study of metformin, its different uses and mechanisms of action were identified. Metformin reduces blood glucose levels mainly by inhibiting hepatic gluconeogenesis,^[1]^ promoting glucose uptake by skeletal muscles,^[2]^ and improving insulin sensitivity.^[3]^ It can improve ischemic brain damage by inhibiting mitochondrial respiratory chain complex 1,^[4]^ regulating mitochondrial function^[5]^ and energy metabolism by activating AMP-activated protein kinase,^[6,7]^ improving cell metabolism and cell senescence in age-related diseases,^[8,9]^ and exerting anti-tumor effects.^[10,11]^ Recent studies have shown that metformin can alter the gut microbiota of the body and has beneficial effects in a variety of diseases.

Gut microbiota is closely related to human health, and its microbial composition changes with age^[12]^ and plays a key role in the immune regulation of intestinal and extraintestinal organs.^[13]^ An imbalance in the intestinal flora leads to weakening of immune regulation and can easily induce many diseases. Several studies have confirmed that metformin improves the gut flora in patients. For example, metformin can change the structure of the intestinal flora in T2DM patients, exert hypoglycemic effects, and regulate blood glucose homeostasis.^[14,15]^ It can inhibit tumor growth by affecting the composition of intestinal flora^[16]^ and regulating the gut microbiota to alleviate sepsis-related liver injury^[17]^ and neuroinflammation.^[18]^ Therefore, it is necessary to conduct a bibliometric study of metformin and the gut microbiota to capture the latest research hotspots and trends.

Bibliometrics is a literature analysis method that uses mathematical and statistical methods to retrospectively review, summarize, and analyze publications in a specific research field and predict future developments.^[19]^ Bibliometrics often relies on bibliometric software, such as VOSviewer, CiteSpace, HistCite, and the “bibliometrix” package in R. These software programs are used to visually analyze publication research, display research results more intuitively, and improve readers’ understanding of research activities. Bibliometric studies have been conducted in many research fields such as diabetes and intestinal flora,^[20]^ pain and intestinal microbes,^[21]^ intestinal microbiota and tumor immunotherapy,^[22]^ and liver glucose and lipid metabolism.^[23]^ However, there are no bibliometric studies on the relationship between metformin and gut microbiota. Recent studies have shown that the regulation of the gut microbiota by metformin can affect an increasing number of diseases. This study conducted a bibliometric analysis of publications on metformin and gut microbiota over the past 11 years (2012–2022), identified the main contributors (including authors, countries, and institutions) and research status, and clarified the research focus and trends in this field.

## 2. Methods

### 2.1. Database sources and search strategies

**Figure 1.**
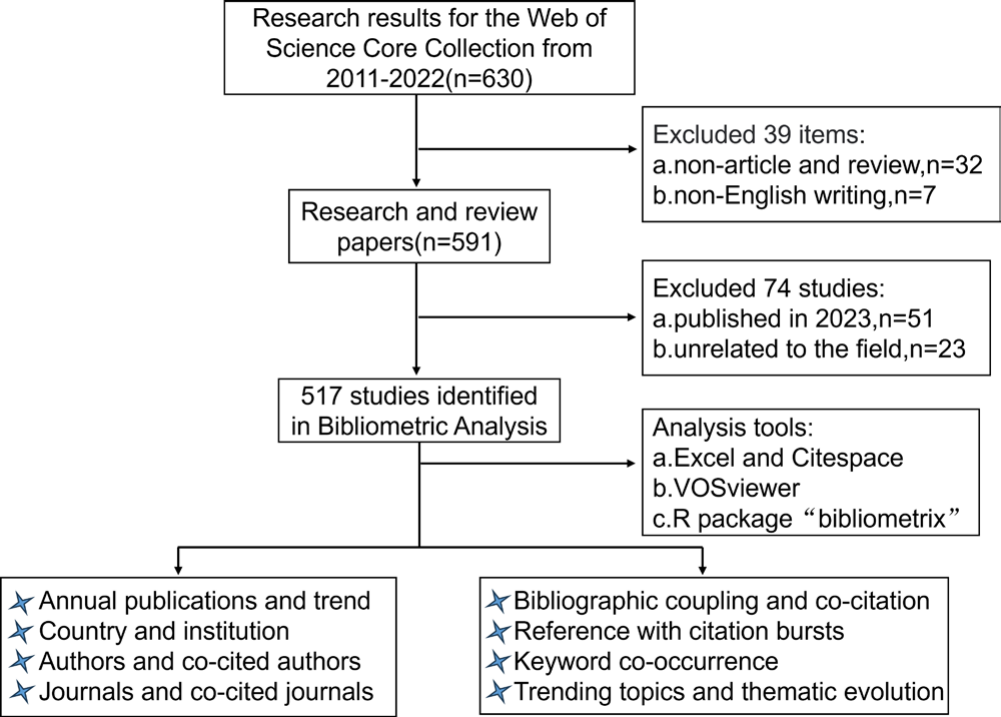
Retrieval flow chart for publications related to metformin and gut microbiota.

The Web of Science (WoS) database is one of the most commonly used and authoritative databases, and the academic authority of the journal papers in its core collection is recognized by the global academic community, containing 12,000 influential, high-quality journals from countries around the world.^[24,25]^ Compared with PubMed and the complete WoS collection, the WoS Core Collection (WoSCC) database has a complete citation index and a large number of bibliometrics indicators, and is widely used in bibliometrics research in different fields.^[24,26–29]^ Relevant publications were obtained by searching the WoSCC database (https://www.webofscience.com/wos/woscc/basic-search) on June 8, 2023. The search formula was: Theme = (metformin) AND Theme=(“Gastrointestinal Microbiome” or “Gastrointestinal Microbiomes” OR “Gut Microbiome” OR “Gut Microbiomes” OR “Gut Microflora” OR “Gut Microbiota” OR “Gut Microbiotas” OR “Gastrointestinal Flora” OR “Gut Flora” OR “Gastrointestinal Microbiota” OR “Gastrointestinal Microbiotas” OR “Gastrointestinal Microbial Community” OR “Gastrointestinal Microbial Communities” OR “Gastrointestinal Microflora” OR “Gastric Microbiome” OR “Gastric Microbiomes” OR “Intestinal Microbiome” OR “Intestinal Microbiomes” OR “Intestinal Microbiota” OR “Intestinal Microbiotas” OR “Intestinal Microflora” OR “Intestinal Flora” OR “Enteric Bacteria”). The literature types were “article” and “review,” the language was “English,” and articles published in 2023 were excluded. To avoid human retrieval errors, this operation was carried out independently by 2 postgraduate students (Yang Shu and Weidong Li) on June 8, 2023. All selected literature records (title, author, institution, country, keywords, and published journal) and cited references were exported in the form of plain text files. We read the titles and abstracts of all the literatures selected from the database one by one through an independent analysis of 2 persons, compared the selected articles, and confirmed the controversial articles after reading the full text.^[23,30]^ Excluding papers that were not part of the metformin and gut microbiota studies, such as “Understanding heterogeneity of responses to, and optimizing clinical efficacy of, exercise training in older adults: NIH NIA Workshop summary.” This article was retrieved, but it did not belong to the study of metformin and gut flora, but to the study of exercise response in the elderly population. This method was also used for other excluded documents. We retrieved 630 articles related to metformin and intestinal bacteria, among which 517 (raw data are presented in the Supplementary Material, http://links.lww.com/MD/K961) articles that met the screening criteria were analyzed using bibliometrics and visualization (Fig. 1).

### 2.2. Data processing and visual analysis

Excel is a powerful data processing and analysis software. In this study, we used Microsoft Excel 2021 (version 2305, Microsoft, Redmond, Washington, USA) to quantitatively and visually analyze the volume of publications in different countries. VOSviewer (version 1.6.19, Leiden University Science and Technology Research Center, Leiden, Netherlands)^[31]^ is bibliometric analysis software commonly used to explore co-authorship, co-occurrence, citations, literature coupling, co-citation presenting network visualization, overlay visualization, density visualization^[32]^ and cluster analysis.^[33]^ In our study, VOSviewer primarily completed visualizations of the national cooperation network, institution overlay, co-cited authors, literature coupling, and keyword co-occurrence. In the visual map generated by VOSviewer, a node represents a research project such as authors, countries, institutions, and references. The node size and color represent the number and clustering of these items, respectively, and the line thickness between nodes reflects the degree of cooperation or co-citation strength between items.^[26]^

CiteSpace (version 6.2.4, Drexel University, Philadelphia, USA)^[34,35]^ is a Java-based application developed by Professor Chao-mei Chen that is also used for bibliometric analysis and visualization. This software can analyze research trends in a certain field and show the research structure of this field in the form of a visual knowledge graph.^[36]^ In this study, CiteSpaceV was used to detect citation bursts. The R package “bibliometrix” (version 4.0.0, Federico II University of Naples, Italy),^[37]^ is a package that must be used in the R environment and is a comprehensive atlas analysis tool. This study used the “bibliometrix” R package to complete the analysis of the countries’ collaboration world map, rectangular tree of keywords, trending topics, and thematic evolution.

### 2.3. Ethics and consent

This study was based on published academic papers, and did not involve animals or humans. Therefore, ethical approval was not required for this study.

## 3. Results

### 3.1. Annual publications and trend

**Figure 2.**
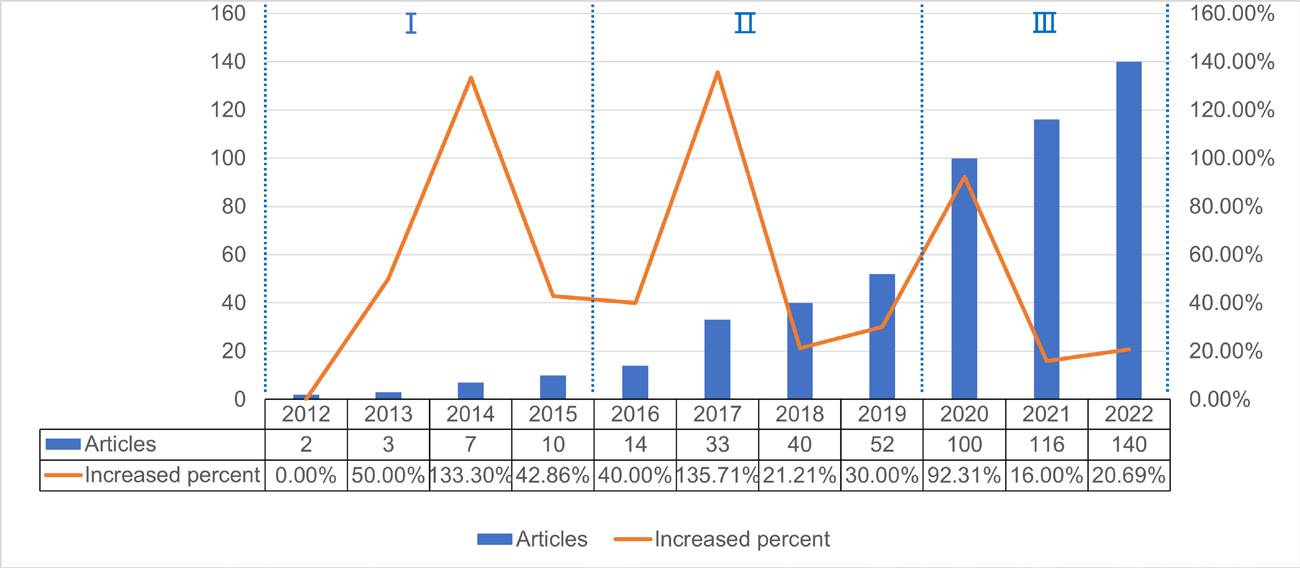
Annual trend and growth rate of publications related to metformin and gut microbiota.

Using our search strategy, we obtained 517 articles on metformin and the gut microbiota from 2012 to 2022, including 377 research articles and 140 review articles. Figure 2 shows the annual volume and growth rate of publications related to metformin and gut microbiota. Publication related to metformin and intestinal flora can be divided into 3 stages, with 3 peaks in annual growth rate. The first stage was from 2012 to 2014; the annual growth rate of this stage increased annually, but the number of papers published each year was <10, indicating that this stage is the initial stage of metformin and intestinal flora related research, and people have begun to pay attention to this field. The second stage occurred from 2015 to 2019, with an annual growth rate of more than 20% and a peak growth rate of 2017 (135.71%). By 2019, the number of articles had reached 52, indicating that the fields of metformin and intestinal flora have aroused the interest of researchers and received more attention, and the literature output is increasing. In the third stage, 2020–2022, the annual number of papers reached more than 100, and reached the peak growth rate in 2020 (92.31%), after which the annual growth rate remained at 16% or more, indicating that metformin and intestinal flora related research is becoming increasingly important, and the field is booming.

### 3.2. Country and institution

A total of 517 articles on metformin and gut microbiota were obtained from 57 countries and included 991 institutions. As shown in Table 1, the top 10 countries engaged in metformin and gut microbiota research published 495 articles, accounting for 95.74% of all the publications. China contributed the most (45.07%, n = 233), followed by the United States (17.6%, n = 91). According to citation analyses, China had the highest number of citations, reaching 6817. This was followed by the USA (4447 citations) and England (3544 citations). As shown in Table 2, the organization with the highest number of publications is the Chinese Academy of Sciences (3.09%, n = 16), followed by Shanghai Jiao Tong University (2.71%, n = 14). A citation analysis revealed that Central South University had 2746 citations. The citations of the University of Gothenburg (1225) and the Chinese Academy of Sciences (881) were lower than those of the Central South University.

**Table 1:** Top 10 productive countries in metformin and gut microbiota studies, 2012 to 2022.

Rank	Country	Counts	% of 517	Citations	Total link strength
1	Peoples R China	233	45.07	6817	54
2	USA	91	17.60	4447	59
3	South Korea	30	5.80	2148	8
4	Canada	26	5.03	780	21
5	England	25	4.84	3544	30
6	France	21	4.06	2781	21
7	Spain	20	3.87	1345	10
8	Italy	19	3.68	648	12
9	Denmark	15	2.90	2769	21
10	Netherlands	15	2.90	896	10

**Table 2:** Top 10 productive organizations in metformin and gut microbiota studies, 2012 to 2022.

Rank	Organization	Counts (%)	Citations	Total link strength
1	Chinese Academy Sciences (China)	16 (3.09)	881	22
2	Shanghai Jiao Tong University (China)	14 (2.71)	880	11
3	Central South University (China)	13 (2.51)	2746	12
4	Sun Yat-sen University (China)	13 (2.51)	216	8
5	University of Copenhagen (Denmark)	13 (2.51)	103	3
6	Harvard Medical School (USA)	10 (1.93)	381	2
7	Beijing University of Chinese Medicine (China)	9 (1.74)	367	11
8	Fudan University (China)	9 (1.94)	354	7
9	University of Gothenburg (Sweden)	9 (1.74)	1225	6
10	University of Toronto (Canada)	9 (1.74)	361	2

Table percentages were calculated by dividing the row count by the total number of publications (n = 517).

**Figure 3.**
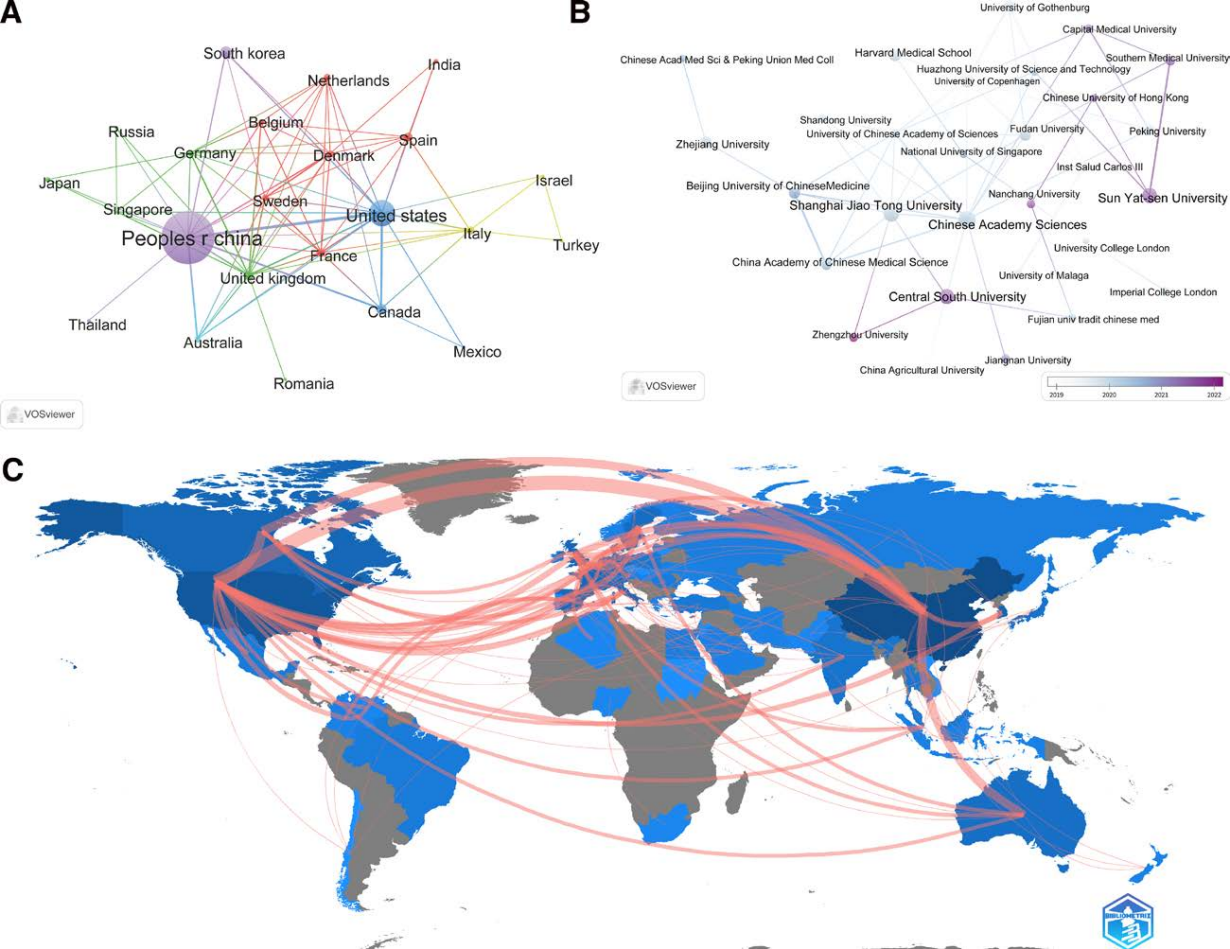
Country and institution analysis of metformin and gut microbiota related publications from 2012 to 2022. (A) Network visualization map of countries’ collaboration by VOSviewer. The minimum number of documents of a country was set as 4, 28 of the 57 countries that participated in the metformin and gut microbiota study met the requirements. (B) Overlay visualization map of main research institutions’ collaboration by VOSviewer. The minimum number of documents of an organization was set as 5, 35 of the 991 organizations that participated in the metformin and gut microbiota study met the requirements. (C) Countries’ collaboration world map by “bibliometrix” R package. The red lines connect different countries or regions, and the width of the lines represents the closeness of cooperation.

We used VOSviewer to build a collaborative network map of countries and institutions (Fig. 3A and B), and the “bibliometrix” R package to create countries’ collaboration world map (Fig. 3C). As shown in Figure 3, China published more articles than the United States; however, the United States was more closely engaged with the international community than China. In terms of institutions, most institutions in China had domestic cooperation, and there was less cooperation with the international community. China and its scientific research institutions must travel abroad to participate in international cooperation.

### 3.3. Authors and co-cited authors

According to the search results, 517 articles contained 3316 authors and 18,807 cited authors who contributed to the field of metformin and gut microbiota. Among all authors, 11 authors have published 5 or more articles, among which *Kim* and *Li* published 6 articles each, and the remaining 9 authors have published 5 articles, indicating that they have contributed to the development of the field. The co-citation of the cited authors’ information was also analyzed. Among all co-cited authors, 11 had more than 100 citations (Table 3). *Cani* (272 citations) ranked first, followed by *Wu* (245 citations) and *Forslund* (228 citations).

**Table 3:** Top 11 authors and co-cited authors in metformin and gut microbiota studies,2012 to 2022.

Rank	Authors	Counts	Rank	Co-cited authors	Citations
1	Li, M	6	1	Cani, PD	272
2	Kim, H	6	2	Wu, H	245
3	Bose, S	5	3	Forslund, K	228
4	Ke, H	5	4	Qin, GG	191
5	Lee, H	5	5	Shin, NR	188
6	Mueller, NT	5	6	Lee, H	158
7	Park, S	5	7	Turnbaugh, PJ	157
8	Tian, J	5	8	De la cuesta-zuluaga, J	146
9	Tinahones, FJ	5	9	Everard, A	142
10	Wang, JH	5	10	Karlsson, FH	134
11	Zhang, T	5	11	Zhang, X	103

**Figure 4.**
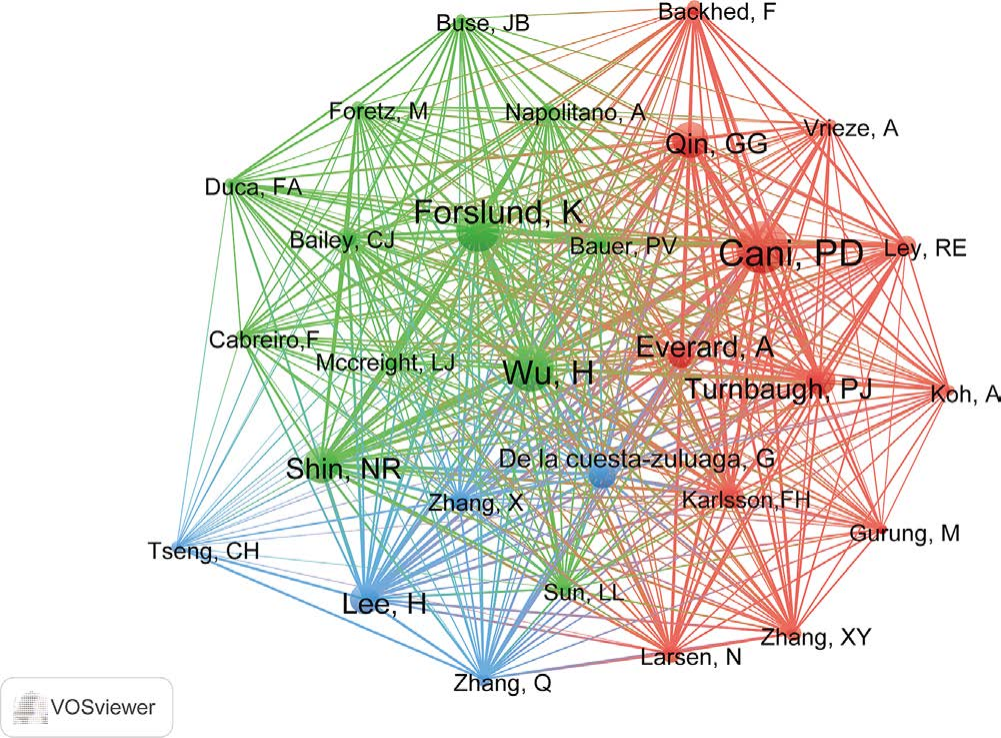
Network of co-cited authors of studies involving metformin and gut microbiota from 2012 to 2022 by VOSviewer. The minimum number of citations of an author was set as 50, 29 of the 18,807 cited authors reached the threshold.

We used VOSviewer to build a network visualization map of the co-cited authors (Fig. 4). The analysis combined with Figure 4 and Table 3 shows that among the top 3 authors, *Wu* (green) and *Cani* (red), *Cani* (red) and *Forslund* (green) were not in the same cluster, *Wu* (green) and *Forslund* (green) were in the same cluster. This showed that *Wu* and *Forslund* have similar or the same research fields, while *Cani* was different from their research fields.

### 3.4. Journals and co-cited journals

In total, 517 articles retrieved for this study were published in 259 journals. Among them, *PLOS ONE* published the most articles (19, 3.68%), followed by *Frontiers in endocrinology* (18, 3.48%), *Frontiers in microbiology* (15, 2.9%), and *Frontiers in pharmacology* (15, 2.9%) (Table 4). These 4 journals published 67 articles on studies related to metformin and gut microbiota, accounting for 12.96% of the 517. We analyzed 3663 co-cited journals by VOSviewer. Table 5 lists the top 10 co-cited journals. *Nature* (1598 citations) ranked first, followed by *PLOS ONE* (1107 citations), *Diabetes Care* (770 citations), and *Nature Medicine* (739 citations). Total citation counts usually reflect the significance of the journal, indicating that in the field of metformin and gut microbiota, *Nature* was the most important and authoritative journal. By combining Tables 4 and 5, we found that studies related to metformin and intestinal flora were mainly published in *PLOS ONE* and *Frontiers* journals, and also found highly cited journals in this field, such as *Nature, Nature Medicine, Cell Metabolism*, all of which are high-level journals.

**Table 4:** Top 10 journals with the most publications on metformin and gut microbiota,2012 to 2022.

Journal	Count	Timespan	IF	JCR
*PLOS ONE*	19 (3.68%)	2014–2022	3.7	Q2
*Frontiers in Endocrinology*	18 (3.48%)	2018–2022	5.2	Q2
*Frontiers in Microbiology*	15 (2.90%)	2015–2022	5.2	Q2
*Frontiers in Pharmacology*	15 (2.90%)	2017–2022	5.6	Q1
*Nutrients*	12 (2.32%)	2017–2022	5.9	Q2
*Biomedicine & Pharmacotherapy*	11 (2.13%)	2018–2022	7.5	Q2
*Frontiers in Cellular and Infection Microbiology*	9 (1.74%)	2020–2022	5.7	Q2
*Scientific Reports*	9 (1.74%)	2015–2022	4.6	Q2
*Diabetologia*	8 (1.55%)	2016–2022	8.2	Q1
*International Journal of Molecular Sciences*	8 (1.55%)	2020–2022	5.6	Q2

IF = impact factors, JCR *=* Journal Citation Reports.

**Table 5:** The 10 most cited journals in the field of metformin and gut microbiota,2012 to 2022.

Cited journal	Citations	Total link strength	IF	JCR
*Nature*	1598	114,996	64.8	Q1
*PLOS ONE*	1107	78,973	3.7	Q2
*Diabetes Care*	770	59,947	16.2	Q1
*Nature Medicine*	739	57,293	82.9	Q1
*Diabetes*	716	63,150	7.7	Q1
*Cell Metabolism*	715	65,135	29.0	Q1
*Diabetologia*	690	55,657	8.2	Q1
*Gut*	667	47,124	24.5	Q1
*Scientific Reports*	627	44,145	4.6	Q2
*PNAS*	619	54,939	11.1	Q1

IF = impact factors, JCR *=* Journal Citation Reports.

### 3.5. Bibliographic coupling and co-citation

**Figure 5.**
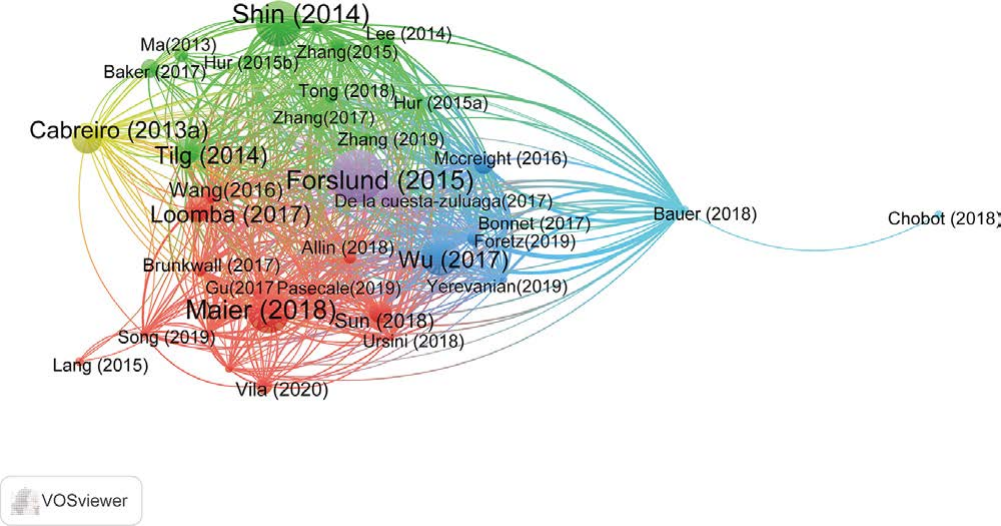
Bibliographic coupling network of publications related to metformin and gut microbiota from 2012 to 2022 by VOSviewer. The minimum number of citations of a document was set as 100, 39 of the 517 documents reached the threshold.

Bibliographic coupling analysis measures the degree of correlation between multiple studies by detecting the same references (Fig. 5). The top ten articles with the most citations in this field are listed in Table 6. As shown in Figure 5 and Table 6, *Forslund Disentangling type 2 diabetes and metformin treatment signatures in the human gut*,^[38]^ published in *Nature* in 2015, had 1219 citations and ranked first. The second was *Shin*, who published an article in *Gut* in 2014 titled “*An increase in the Akkermansia spp. population induced by metformin treatment improves glucose homeostasis in diet-induced obese mice*.”^[39]^ This article has 1018 citations. In addition, from Figure 5, Forslund (purple) and Shin (green), although belonging to different clusters, have both advanced the field with their studies related to metformin and gut microbiota. In the reference co-citation analysis, 517 papers included 24,398 references. Table 7 lists the top 10 most co-cited references, and we find that the 2 studies published by *Forslund* (2015) in *Nature medicine* and *Wu* (2017) in *Nature* were both co-cited 223 times. Therefore, through bibliographic coupling and co-citation analysis, we can find all the information about the most relevant and authoritative literature in the field.

**Table 6:** Top 10 most citing articles globally in the field of metformin and gut microbiota, 2021 to 2022.

Citing paper	Yr	Journals	Country	First author	Citations
Disentangling type 2 diabetes and metformin treatment signatures in the human gut microbiota	2015	Nature	Germany	Forslund, K	1219
An increase in the *Akkermansia* spp. population induced by metformin treatment improves glucose homeostasis in diet-induced obese mice	2014	Gut	Korea	Shin, NR	1018
Extensive impact of non-antibiotic drugs on human gut bacteria	2018	Nature	Germany	Maier, L	916
Metformin alters the gut microbiome of individuals with treatment-naive type 2 diabetes, contributing to the therapeutic effects of the drug	2017	Nature medicine	Sweden	Wu, H	860
Metformin retards aging in *Caenorhabditis elegans* by altering microbial folate and methionine metabolism	2013	Cell	UK	Cabreiro, F	616
Gut microbiome-based metagenomic signature for noninvasive detection of advanced fibrosis in human nonalcoholic fatty liver disease	2017	Cell metabolism	USA	Loomba, R	508
Microbiota and diabetes: an evolving relationship	2014	Gut	Austria	Tilg, H	492
Gut microbiota and intestinal FXR mediate the clinical benefits of metformin	2018	Nature medicine	China	Sun, L	437
Metformin is associated with higher relative abundance of mucin-degrading *Akkermansia muciniphila* and several short-chain fatty acid–producing microbiota in the gut	2017	Diabetes care	Colombia	De la cuesta-zuluaga, J	392
Metformin and the gastrointestinal tract	2016	Diabetologia	UK	Mccreight, LJ	368

**Table 7:** Top 10 co-cited reference in the field of metformin and gut microbiota,2012 to 2022.

Rank	Co-cited reference	Citations
1	Forslund K, 2015, NATURE, V528, P262. DOI 10.1038/nature15766	223
2	Wu H, 2017, NAT MED, V23, P850. DOI 10.1038/nm.4345	223
3	Qin GG, 2012, NATURE, V490, P55. DOI 10.1038/nature11450	165
4	Shin NR, 2014, GUT, V63, P727. DOI 10.1136/gutjnl-2012-303839	160
5	De la cuesta-zuluaga G, 2017, DIABETES CARE, V40, P54. DOI 10.2337/dc16-1324	142
6	Karlsson FH, 2013, NATURE, V498, P99. DOI 10.1038/nature12198	124
7	Lee H, 2014, APPL ENVIRON MICROB, V80, P5935. DOI 10.1128/aem.01357-14	110
8	Larsen N, 2010, PLOS ONE, V5. E9085. DOI 10.1371/journal.pone.0009085	95
9	Turnbaugh PJ, 2006, NATURE, V444, P1027. DOI 10.1038/nature05414	83
10	Everard A, 2013, P NATL ACAD SCI USA, V110, P9066. DOI 10.1073/pnas.1219451110	81

### 3.6. Reference with citation bursts

**Figure 6.**
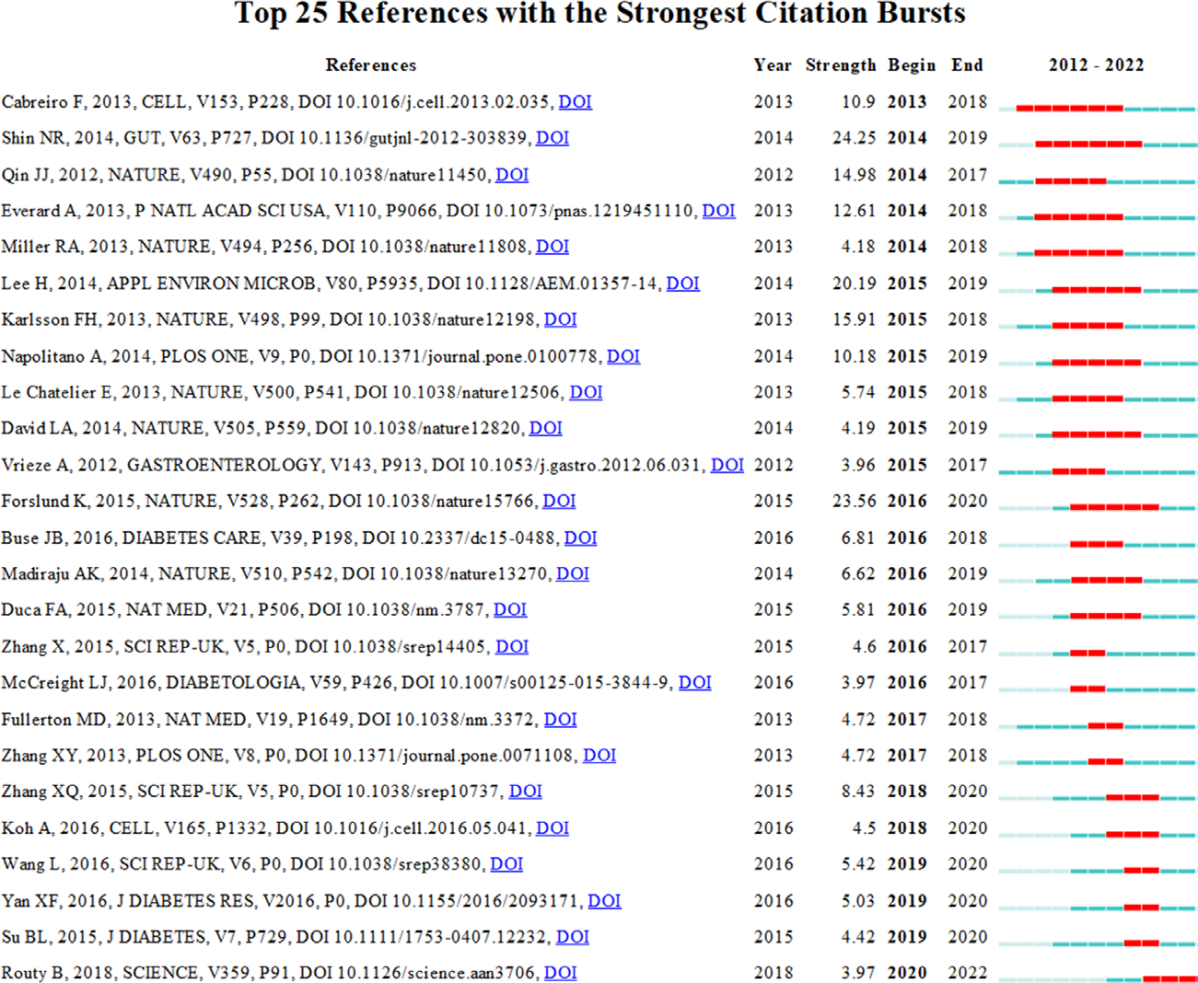
Top 25 References with the Strongest Citation Bursts by CiteSpace. Each bar in the figure represents the yr, and the red bar represents the yr with high citations.

Citation burst refers to literature frequently cited by scholars in the field over time. In this study, the 25 articles with the strongest citation bursts were screened using CiteSpace. Figure 6 shows a list of burst features that visually presents representative literature from different periods. Each bar in the figure represents the year, and the red bar represents the year with high citations,^[40]^ indicating that a certain piece of literature received special attention during this period. Citation bursts for references in this field first appeared in 2013 and have occurred every year since then, until 2022. The reference with the strongest citation burst (strength = 24.25) was Shin et al ’s article in *Gut* (2014). The citation burst period in this study was 2014 to 2019. Forslund et al in *Nature* in 2015 had the second strongest citation burst (strength = 23.56) and the citation burst period was from 2016 to 2020. Overall, the burst strength of these 25 references ranged from 3.96 to 24.25, and the endurance strength was 2 to 6 years (Fig. 6).

### 3.7. Keyword co-occurrence

**Figure 7.**
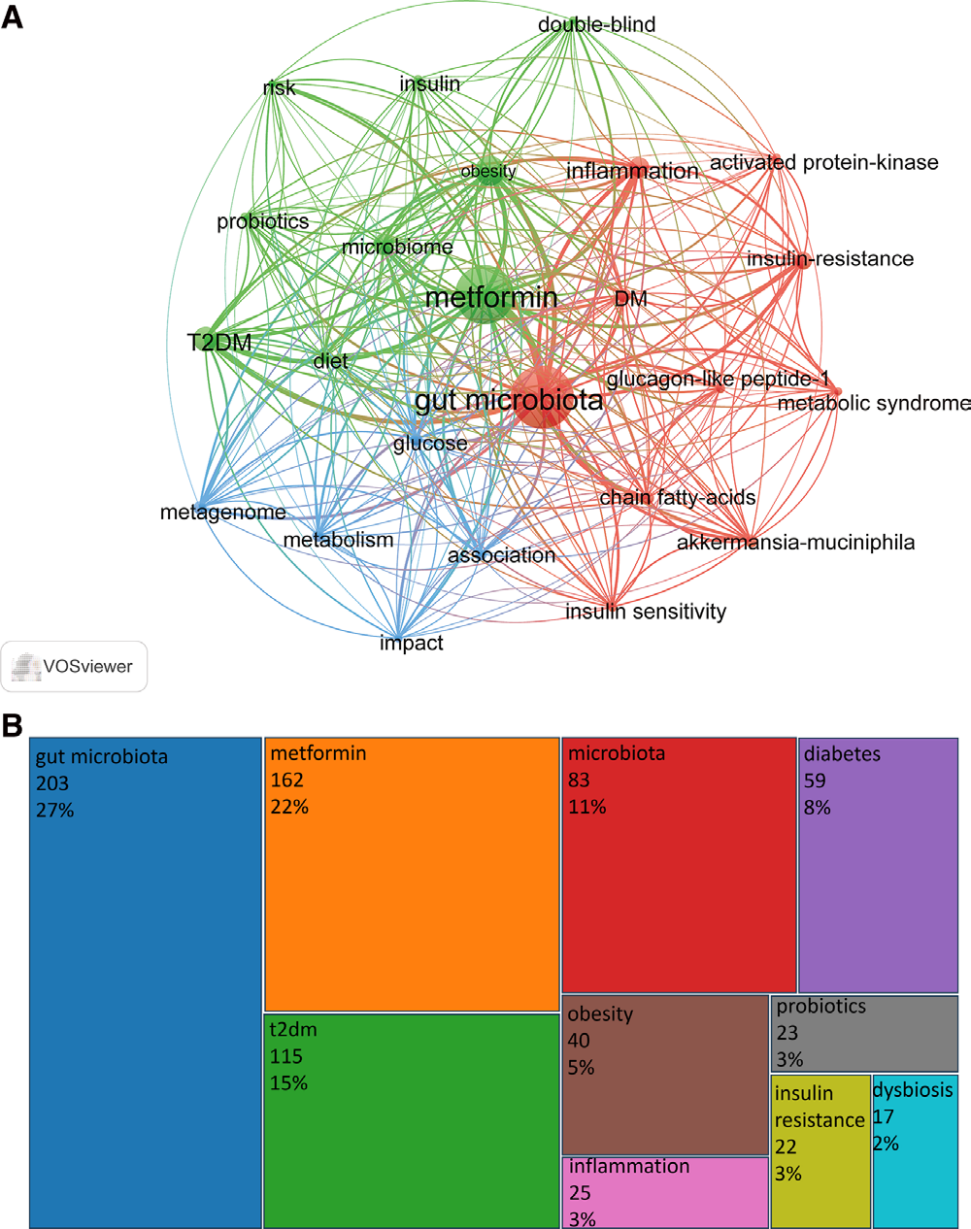
Keywords co-occurrence analyses in the field of metformin and gut microbiota. (A) Network visualization map of author keywords by VOSviewer. The minimum co-occurrence of author keywords was set to 10, and 24 articles met the requirements. (B) Rectangular tree with keyword co-occurrence by “bibliometrix” R package. The different colors of the rectangle represent different keywords, and the size of the rectangle represents the number and percentage of co-occurrence of keywords. The more the co-occurrences, the larger the area of the rectangle.

The purpose of co-occurrence analysis is to identify the co-occurrence relationships of keywords in the literature. If a document simultaneously contains multiple keywords, a co-occurrence relationship exists between these keywords. Co-occurrence analysis can help reveal thematic associations within a research field, identify popular keywords, and understand the structure of the research field. To avoid repetition of keywords with the same or similar meaning affecting the calculation and drawing, we adopted the method of synonym merging. We used VOSviewer to analyze the author keywords for 517 articles and obtained 1069 author keywords (Fig. 7A). We used the “bibliometrix” R package to draw a rectangular tree diagram of keywords. Figure 7B shows the top ten keywords with highest number of co-occurrences and their relative proportions. In addition to metformin and gut microbiota, T2DM, microbiome, diabetes, obesity, inflammation, probiotics, insulin resistance, and dysbiosis are also frequent in this field. These keywords were closely related to the occurrence and treatment of metabolic diseases such as DM. Metformin and intestinal flora may be mainly used in the study of metabolic diseases such as T2DM.

### 3.8. Trending topics and thematic evolution

**Figure 8.**
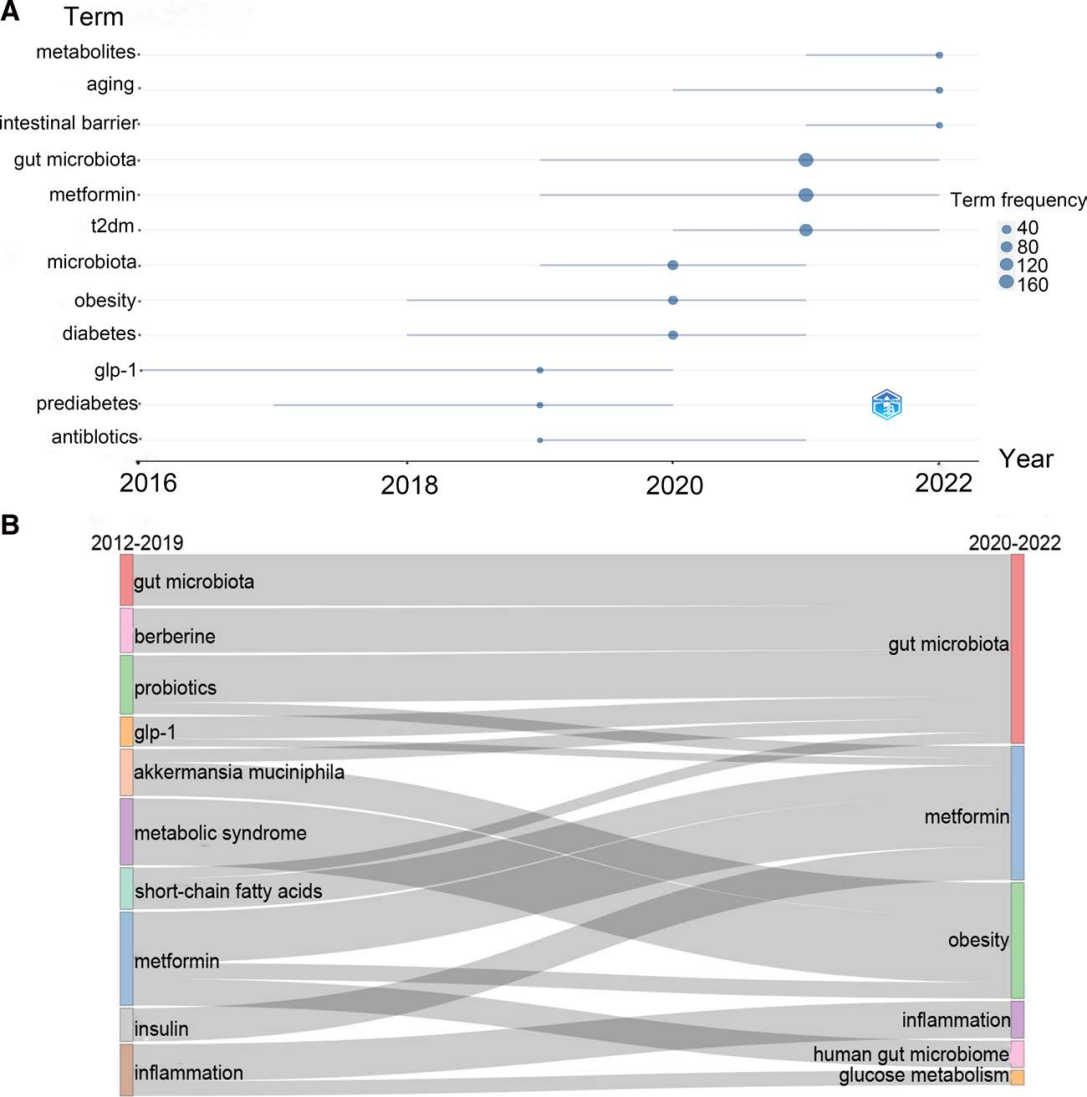
Trending topics and thematic evolution in the field of metformin and gut microbiota. (A) Trending topics graph by “bibliometrix” R package. The size of the circle represents the frequency of the keyword, the position of the circle corresponds to the yr of the median frequency, and the left and right ends of the blue line segment represent the positions corresponding to frequencies of 25% and 75%, respectively. (B) Thematic evolution graph by “bibliometrix” R package. The gray lines represent the theme evolution route, and the rectangles of different colors on the left and right sides represent different themes.

The trend topic and thematic evolution of keywords can be used to analyze the research focus in recent years, future research trends, and topic changes in this field. Figure 8A shows the prevalence trends of the keywords in recent years. It can be seen that gut microbiota, metformin, and T2DM appeared frequently, indicating that these keywords were the hotspots of research in the past 3 years (2020–2022). “Metabolites,” “aging,” and “intestinal barrier” were new popular topics in this field (2021–2022). In thematic evolution analysis, to ensure that metformin and intestinal flora always appear in the topic, the Louvain algorithm was used for topic segmentation and clustering, which is a heuristic clustering algorithm based on modularity optimization^[41,42]^ and has been widely used in medical research.^[43,44]^ Figure 8B shows the theme evolution process from 2012 to 2022. In 2012 to 2019, the most studied topics were metformin and metabolic syndrome, followed by gut microbiota, but in 2020 to 2022, they were gut microbiota and metformin. Topics such as berberine, probiotics, glp-1, and *Akkermansia muciniphila* flowed to the gut microbiota, whereas topics such as probiotics, short-chain fatty acids, and insulin flowed to metformin, indicating that the gut microbiota and metformin evolved from multiple themes and are major research topics in this field.

## 4. Discussion

In this study, we used Excel, VOSviewer, CiteSpace, and R software “bibliometrix” to analyze the publication and research trends of metformin and gut microbiota from many aspects, systematically showed research in this field over the past 11 years, and provide guidance for future research. Therefore, researchers interested in metformin and gut microbiota can use our bibliometric analysis to quickly understand the current research status and identify the latest research hotspots in this field.

In terms of countries and institutions. China not only published the most articles but also cooperated closely with the United States, Canada, Australia and other countries. Although the United States had fewer publications than China, it had more international cooperation than China, such as India, Spain, and Mexico, which did not cooperate with China (Table 1 and Fig. 3A and C). In the institutional analysis, the Chinese Academy of Sciences published the most papers and cooperated closely with many domestic institutions, but cooperated less with foreign countries, and the number of citations was less than that of Central South University (Table 2 and Fig. 3B). Through a comprehensive analysis of countries and institutions, we found that China and the United States have made positive contributions to the field of metformin and gut microbiota, and China has more scientific research institutions to promote the development of this field. However, China and its scientific research institutions were not enough to participate in international cooperation, which required China and its scientific research institutions to go abroad and participate more in international cooperation and exchanges.

For the author analysis, we identified the authors with the most published and co-cited articles. *Cani* is the most frequently cited author, with 272 citations, indicating that *Cani* has in-depth and authoritative research in this field. We carefully reviewed the research areas of *Cani* and identified intestinal flora,^[45,46]^ microbiota,^[47,48]^ and metabolic diseases, such as obesity^[49]^ as the major research areas. He has published a number of articles in high-level journals such as *Nature Medicine, Nature Reviews, Gastroenterology & Hepatology*, and *Gut*, which further shows that *Cani* is a very influential author in the field of intestinal flora research, and his research has made a great contribution to the development of the field.

By analyzing the journals, we found the most published and cited journals in the field (Tables 3 and 4). Of the top 10 journals, 76 papers were published in *PLOS ONE* and 4 *Frontiers (endocrinology, microbiology, pharmacology, cellular and infection microbiology*) journals. This accounted for 14.7% of the total number of publications, indicating that *PLOS ONE* and the *Frontiers* series journals were mainstream journals in the field of metformin and gut microbiota. Among all the co-cited journals, *Nature* was the most co-cited journal, with 1598 citations and a total link strength of 114,996 (Table 5), much higher than *PLOS ONE*. Analyses of the journals showed that in the fields of metformin and gut microbiota, although there were fewer articles published in *Nature, Nature Medicine, Cell Metabolism*, and *Gut* than those published in *PLOS ONE* and Frontier journals, their influence and authority were far higher.

Of the 517 articles included in this study, the most cited paper (Table 6)—“*Disentangling type 2 diabetes and metformin treatment signatures in the human gut microbiota*”—was published in *Nature*.^[38]^ This was followed by “An increase in the *Akkermansia* spp. population induced by metformin treatment improves glucose homeostasis in diet-induced obese mice” was published in *Gut*.^[39]^ These 2 articles had citation bursts in 2016 to 2020 and 2014 to 2019 respectively, demonstrating their importance and influence of these 2 articles in this field. Moreover, in our citation burst analysis, we found that 3 articles published in *Gut* (impact factors [IF] 24.5), *Nature* (IF 64.8), and *Appl Environ Microbiol* (IF 4.4) had citation burst strengths >20. This shows that journal with high or low IF are not the determinant factors of citation bursts, but the quality of the article itself is related to strong citation bursts.

We conducted co-occurrence, trending topics, and thematic evolution analyses of keywords from 517 articles using VOSviewer and the “bibliometrix” R package. Our analysis showed that in addition to the 2 keywords “metformin” and “gut microbiota,” T2DM, microbiome, diabetes, obesity, inflammation, probiotics, insulin resistance, and dysbiosis were also frequent keywords in this field. Some of these keywords also appeared in the analysis of trending topics, such as gut microbiota, metformin, T2DM, microbiome, diabetes, and obesity (Fig. 8A). This suggests that high-frequency keywords were likely to be a hot topic during this period. In addition, some new trends have emerged in this field, including “metabolites,” “aging,” and “intestinal barrier,” which provides new hotspots and directions for this field. Future studies may involve metformin affecting metabolites, aging, and the intestinal barrier through gut microbiota.

In summary, we have drawn several conclusions from the literature on metformin and the gut microbiota from 2012 to 2022. Metformin, an old drug for the treatment of T2DM,^[46,50]^ not only has hypoglycemic effects, but also has the effects of anti-aging,^[51]^ weight loss,^[52]^ cardiovascular protection,^[53]^ improvement of polycystic ovary syndrome,^[54]^ tumor inhibition,^[55,56]^ anti-inflammation,^[57]^ reversal of pulmonary fibrosis^[58]^ and reversal of cognitive dysfunction.^[59]^ In addition, metformin can improve intestinal flora, restore the proportion of intestinal flora, and play a positive regulatory role in the intestinal immune system.^[60,61]^ In studies on obese and T2DM patients, metformin significantly affected the proportion of some gut microbes, such as *Bacteroidetes, Verrucomicrobia, Akkermansia, Bacteroides*, and *Escherichia*.^[62]^ In randomized trials in overweight/obese adults who had been treated for solid tumors but not for cancer, metformin treatment increased *Escherichia coli* and *Ruminococcus torques*, decreased *Intestinibacter bartlettii* and the genus *Roseburia*, including *Roseburia faecis* and *Roseburia intestinalis*.^[63]^ However, some studies have indicated that changes in the intestinal flora caused by metformin may cause gastrointestinal intolerance,^[38,64,65]^ and that metformin combined with microbiome modulators can improve gastrointestinal symptoms.^[66]^ Therefore, the alteration of gut microbiota by metformin is a “double-edged sword” that can produce either beneficial or harmful effects.

## 5. Conclusion

Overall, we conducted a comprehensive bibliometric analysis of the effects of metformin on gut microbiota. This study reviewed the pleiotropy of metformin in the treatment of different diseases and focused on its beneficial effects on diseases by regulating the gut microbiota, which is a current research hotspot and trend in the field. Through multi-dimensional analysis of countries, institutions, authors, journals, literature and keywords, it helps us to understand the cooperation between different countries and institutions, and find the most influential authors, the most authoritative journals and literatures, and also discover emerging topics in the field, such as “metabolites,” “aging,” and “intestinal barrier.” The emergence of new topics indicates a method for follow-up research. It is hoped that this study will provide useful guidance for researchers in the field of metformin and gut microbiota and that the effect of metformin on gut microbiota can be paid attention to and studied in more diseases.

## 6. Limitations

Our study has certain limitations. First, all analyzed data were extracted only from the WoSCC database and were not included in the records of other important search engines such as Scopus, PubMed, Embase, and Google Scholar. Second, our study focused only on papers published in English, resulting in omission of articles published in other languages. Finally, we included only research and review articles, and did not consider conference papers or other source articles, which may have led to incomplete inclusion.

## Acknowledgments

We are grateful to the Chengdu Science and Technology Bureau (grant number: 2021-YF05-01726-SN) for supporting this study.

## Author contributions

**Conceptualization:** Daqian Xiong.

**Data curation:** Yang Shu, Weidong Li.

**Funding acquisition:** Qiongying Hu.

**Investigation:** Qiongying Hu.

**Methodology:** Qiongying Hu.

**Software:** Yang Shu, Weidong Li.

**Supervision:** Daqian Xiong.

**Visualization:** Yang Shu, Weidong Li.

**Writing – original draft:** Yang Shu.

**Writing – review & editing:** Yang Shu, Qiongying Hu, Daqian Xiong.

## Supplementary Material


